# Properties of Rice-Based Beverages Fermented with Lactic Acid Bacteria and *Propionibacterium*

**DOI:** 10.3390/molecules27082558

**Published:** 2022-04-15

**Authors:** Patrycja Cichońska, Anna Ziębicka, Małgorzata Ziarno

**Affiliations:** Department of Food Technology and Assessment, Institute of Food Science, Warsaw University of Life Sciences-SGGW (WULS-SGGW), 02-787 Warsaw, Poland; aziebicka@op.pl (A.Z.); malgorzata_ziarno@sggw.edu.pl (M.Z.)

**Keywords:** milk substitutes, propionic acid, lactic acid, starter cultures, bacteria population, texture properties, syneresis, carbohydrates

## Abstract

In recent times, consumers have shown increasing interest in plant substitutes for fermented dairy products. This study aimed to investigate the properties of yogurt-type rice-based beverages fermented with lactic acid bacteria and *Propionibacterium*. The changes in pH, viable population of bacteria, physical properties, and carbohydrate content of these beverages were tested. Fermentation using only *Propionibacterium* was insufficient to obtain a product with an acidity level similar to that of milk-based yogurt (pH < 4.5). After fermentation, the tested beverages had a high number of *Lactobacillus* sp. (7.42–8.23 log10 CFU/mL), *Streptococcus thermophilus* (8.01–8.65 log10 CFU/mL), and *Bifidobacterium animalis* subsp. *lactis* (8.28–8.50 log10 CFU/mL). The hardness (2.90–10.40 N) and adhesiveness (13.79–42.16 mJ) of the samples after 14 days of storage at 6 °C varied depending on the starter culture used. The syneresis of all samples ranged between 29% and 31%, which was lower or close to that of milk-based yogurts. The content of individual sugars in the samples also varied depending on the starter culture used for fermentation. The results suggest that the combination of lactic and propionic fermentation helps in the production of rice-based yogurt-type milk substitutes.

## 1. Introduction

Fermentation, one of the oldest food preservation processes, is used in the processing of many food products. Fermented milk products, cereals, fruits, vegetables, legumes, tubers, meat products, and fish products are consumed worldwide [1,2]. Lactic acid bacteria (LAB) are the main microorganisms used in food fermentation. Species of LAB belong to numerous genera under the family of *Lactobacillaceae* and include bacteria such as *Lacticaseibacillus*, *Lactiplantibacillus*, *Levilactobacillus*, *Lactococcus*, *Leuconostoc*, *Pediococcus*, and *Streptococcus*. They are Gram-positive and typically nonmotile, nonspore-forming rods and cocci. All LAB are anaerobic, and some tolerate low levels of oxygen in the environment [3,4,5]. The most well-known preservative characteristic of LAB is their ability to produce acid, which in turn results in antimicrobial activity. LAB are widely used in fermentation as they enhance food safety, improve organoleptic attributes, enrich nutrients, increase health benefits, and generate flavor and texture [6,7,8]. The metabolic activities of LAB are associated with the production of many beneficial compounds such as organic acids, antimicrobial compounds (so-called bacteriocins), and unique enzymes that can break down complex organic compounds into simple and functional ones [1].

Further, some species of the genus *Propionibacterium* are used in the production of certain types of fermented foods. *Propionibacteria*, which belong to the *Actinobacteria* class, are mesophilic, Gram-positive, nonspore-forming, and anaerobic to aerotolerant rods. *Propionibacteria* can be divided into two groups: “dairy or classical” and “cutaneous”. The cutaneous type (e.g., *Propionibacterium acidifaciens*, *Propionibacterium acnes*) come from the skin/intestine of humans and animals [9], while the classical type (e.g., *Propionibacterium freudenreichii*, *Propionibacterium jensenii*) are generally isolated from milk and dairy environments. The latter are mainly used in industrial applications and fermentation and are often referred to as propionic acid bacteria (PAB) [9,10]. *Propionibacteria* metabolize different carbohydrates (glucose, galactose, lactose, fructose), alcohols, and organic acids and produce propionic and acetic acids and carbon dioxide as the final products [9]. *Propionibacterium* spp. can also produce a variety of biological compounds that enhance human health such as folic acid, proline, trehalose, conjugated linoleic acid, and vitamin B12 and synthesize different bioprotective compounds, such as bacteriocins and antifungal compounds [11,12,13]. In the food industry, *P. freudenreichii* is most often used, mainly as a vitamin producer, biopreservative, cheese-ripening starter, and probiotic [13,14,15]. Its consumption modulates the gut microbiota, for example, by enhancing the *Bifidobacteria* population [16]. PAB are characterized by the ability to produce propionic acid and carbon dioxide, unlike LAB which mainly produce lactic acid during fermentation [17]. As a result, *Propionibacteria* are considered to be important for ripening Swiss-type cheese, wherein they create holes and give a specific taste and aroma [17]. In the dairy industry, these bacteria are not commonly used in the production of fermented yogurt-type products. Yerlikaya et al. [18] investigated the effect of *Propionibacterium shermanii* subsp. *freudenreichii* on probiotic yogurt-type fermented beverages. They observed that PAB, despite being associated mainly with the production of cheese in recent years, can be used in the production of fermented milk-based beverages.

The most popular group of fermented products is fermented dairy [19]. In recent years, consumers have shown increasing interest in plant substitutes for fermented dairy products [20,21]. This is due to factors such as aversion to animal cruelty, desire for a healthy lifestyle, and environmental awareness [22]. Apart from direct consumption, plant-based beverages can also be used as raw materials to develop yogurt-type products [23]. Plant-based beverages are most often described as products obtained by the liquid extraction of shredded plant materials, in the form of colloidal suspensions or emulsions [24]. These products can be made from a variety of raw materials, such as cereals (e.g., oats, rice, millet), seeds (e.g., sesame, hemp), legumes (e.g., soybean, peas), nuts (e.g., almonds, hazelnuts), and others (e.g., coconut) [25,26,27,28].

Cereals have the most potential in the production of nondairy probiotic foods [29]. They are nutrient sources that, along with beneficial physiological effects, can stimulate the growth of *Lactobacilli* and *Bifidobacteria* [29,30]. Among the plant-based beverages made from cereals, rice-based beverages are common; they are rich in carbohydrates and contain more sugars (9.1–27.0 g/100 g) and less protein (0.1–0.8 g/100 g) than cow’s milk (approximately 5.0 g/100 g of sugars and 3.4 g/100 g of protein) [24]. The processing of rice-based beverages leads to the breakdown of carbohydrates into sugars, which gives the product a characteristic sweet taste without the addition of sugars [31,32]. Plant-based milk substitutes can be fermented to produce dairy-free yogurt-type products while rendering the raw material into a more palatable form [20]. Fermentation can help improve the sensory profile, nutritional properties, texture properties, and microbial safety of plant-based milk alternatives [33]. Most fermented plant-based beverages reported in the literature have been developed using bacteria from the *Bifidobacterium*, *Lactobacillus*, and *Streptococcus* genera [23,24]. However, the effect of combining LAB and *Propionibacterium* fermentation on the properties of rice-based beverages has not been investigated so far. Therefore, this study aimed to investigate the properties of rice-based beverages fermented using LAB and *Propionibacterium*. To determine the effect of the fermentation process, the changes in pH and viable population of bacteria in the beverages were tested before fermentation, after fermentation, and after 14 and 28 days of storage. The physical properties of the fermented beverages, including their hardness, adhesiveness, and syneresis, as well as the changes in the carbohydrate content (fructose, glucose, maltose, sucrose, raffinose), were examined after 14 and 28 days of storage.

## 2. Results and Discussion

### 2.1. Active Acidity and Microflora Population

During the fermentation of food using LAB, the pH value decreases, and consequently, the food acquires unique organoleptic properties. The individual characteristics of the products obtained via fermentation (including acidity) should remain at the same level during storage. Therefore, active acidity (pH) and viable microflora population of the studied rice-based beverages were investigated before fermentation, after fermentation, and after 14 and 28 days of storage at 6 °C.

**Figure 1:**
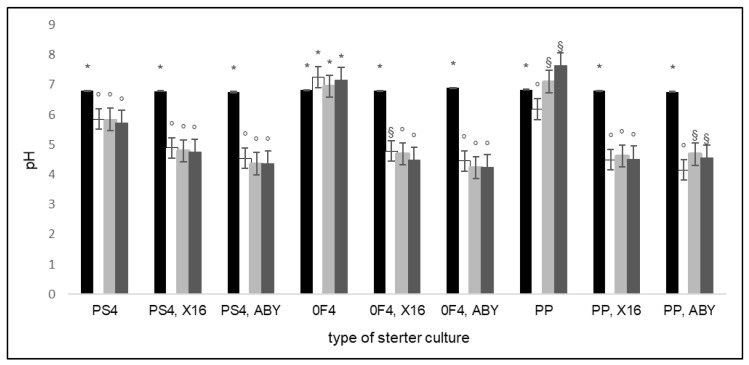
The changes in the pH of rice-based beverages before fermentation (black column), after fermentation (white column), after 14 days of storage (light grey column), and after 28 days of storage (dark grey column) when inoculated with different starter cultures. Within each starter cultures group means with a common symbol are not significantly different (*p* ≥ 0.05). Error bars represent the standard error of the mean.

The analysis showed that the time of storage (*p* < 0.001) and type of starter cultures (*p* < 0.001) significantly influenced the active acidity in the tested beverages. A significant interaction effect of the time of storage × type of starter cultures on the active acidity of the beverages was noted (*p* < 0.001). In the samples fermented with starter cultures containing only *P. freudenreichii* subsp. *shermanii* (PS4, F04, PP), there was no or slight decrease in pH, both after fermentation and during storage. In the samples where the starter cultures contained LAB (X16, ABY), a significant decrease in pH was observed after fermentation, which remained at a similar level during storage. The greatest decrease in pH was observed in samples fermented with the ABY starter culture. The changes in the pH of the rice-based beverages before fermentation, after fermentation, and after 14 and 28 days of storage when inoculated with different starter cultures are presented in Figure 1.

Mojka [34] claims that the pH of fermented yogurt milk drinks should be between 4.0 and 4.5. In the present study, such values were obtained only for the rice-based beverages fermented with cultures of PAB in combination with ABY yogurt starters. The pH of the beverages whose starters contained *Propionibacterium* and the X16 yogurt starter culture were higher and ranged between 4.5 and 4.9. The pH values of rice-based beverages fermented only with *Propionibacterium* (PS4, 0F4, and PP) were higher and reached values between 5.9 and 7.3. These dependencies may be due to the commensalism between PAB and LAB. The latter produce lactic acid, which serves as a preferred carbon source for the former to grow and produce propionic and acetic acid. The nutritional and physical growth requirements of these two microorganisms are complex, and additional LAB and PAB metabolites might support the synergistic growth effects. The interaction varies depending on the particular strain of microorganisms. These interactions are complex but are considered to be crucial for achieving the desired product characteristics [35].

In the present study, most of the tested samples showed no statistically significant changes in the pH value during storage when compared with the samples after fermentation. A slight increase in pH during the 14- and 28-day storage was observed in samples fermented using the PP starter culture and the combination of PP and ABY starter cultures. Bernat et al. [36] investigated the effect of 28-day refrigerated storage of fermented oat yogurt drinks on the pH and bacterial count of *Limosilactobacillus reuteri* (previously classified as *Lactobacillus reuteri*) and *Streptococcus thermophilus*. In their study, in contrast with the results obtained for rice-based beverages, a significant reduction in the pH of oat beverages was observed during storage, from 4.3 to 3.3. There was also a significant reduction in the bacterial count from 8.8 log CFU/mL for *L. reuteri* and 8.1 log CFU/mL for *S. thermophilus* after fermentation to 7.4 log CFU/mL for *L. reuteri* and 7.6 log CFU/mL for *S. thermophilus* after 28 days of storage. Maintaining the pH and bacterial count obtained after fermentation at a constant level throughout the storage period is important. This allows the product to have constant organoleptic characteristics and a sufficiently high number of bacteria beneficial to health.

The samples of rice-based beverages were assessed for the viable population of microflora, declared by the producer as present in the used starter cultures. The study examined whether the microbial population obtained after fermentation remained at a similar level after 28 days of storage. The analysis showed that the time of storage (*p* < 0.001) and type of starter cultures (*p* < 0.001) significantly influenced the viable population of *P. freudenreichii* subsp. *shermanii*, *Lactobacillus* sp., *S. thermophilus*, and *Bifidobacterium animalis* subsp. *lactis*. In addition, there was a significant interaction effect (*p* < 0.001) of the time of storage × type of starter cultures on the viable population of these microorganisms. In the case of *Lactobacillus acidophilus*, the time (*p* < 0.001) and type of starter cultures (*p* = 0.006) significantly influenced the viable population of these microorganisms, but there was no significant interaction effect (*p* = 0.051).

**Figure 2:**
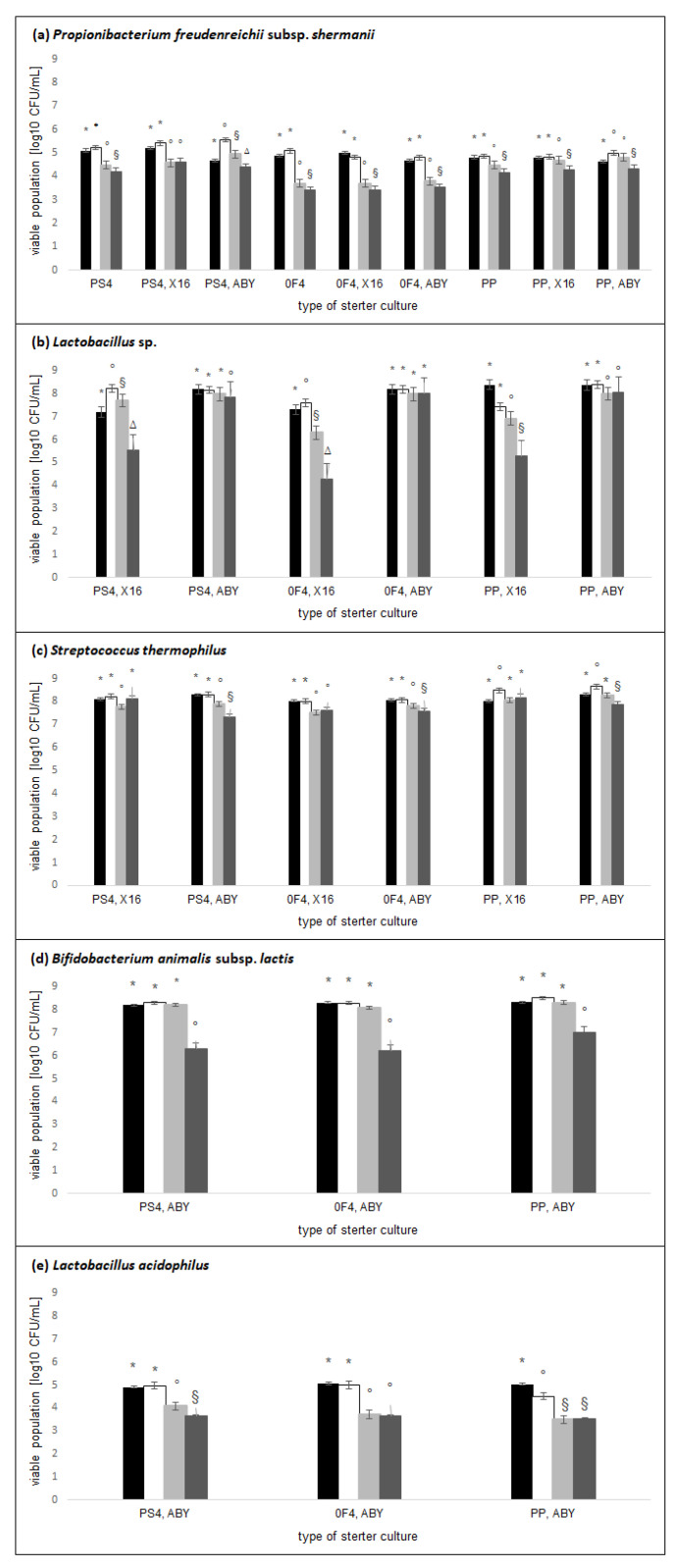
The changes in the viable bacteria population of Propionibacterium freudenreichii subsp. shermanii, Lactobacillus sp., Streptococcus thermophilus, Bifidobacterium animalis subsp. lactis, and Lactobacillus acidophilus in rice-based beverages before fermentation (black column), after fermentation (white column), after 14 days of storage (light grey column), and after 28 days of storage (dark grey column) when inoculated with different starter cultures. Within each starter culture, the group means with a common symbol are not significantly different (*p* ≥ 0.05). Error bars represent standard error of the mean.

The viable population significantly differed depending on the tested microorganisms, which is presented in Figure 2. The highest count of the viable population after fermentation was obtained for *Lactobacillus* sp. (7.42–8.23 log10 CFU/mL), *S. thermophilus* (8.01–8.65 log10 CFU/mL), and *B. animalis* subsp. *lactis* (8.28–8.50 log10 CFU/mL). A significantly lower count was obtained for *P. freudenreichii* subsp. *shermanii* (4.80–5.56 log10 CFU/mL) and *L. acidophilus* (4.52–5.00 log10 CFU/mL). In most of the tested samples, a significant decrease in the viable population was observed after 14 days of storage. The count of the viable population of *P. freudenreichii* subsp. *shermanii*, *B. animalis* subsp. *lactis*, and *L. acidophilus* decreased significantly during the 28-day storage period in all of the tested samples. However, there was no significant reduction in the viable population of *Lactobacillus* sp. in samples fermented with the combination of 0F4 and ABY starter cultures and of *S. thermophilus* in samples fermented with PS4–X16 or PP–X16 combinations.

According to the definition provided by the Food and Agriculture Organization of the United Nations and World Health Organization [37], the microorganisms incorporated into yogurt should be in abundance and of high viability. The sum of the microorganisms constituting the starter culture should be at least 7 log10 CFU/mL. In the tested rice-based beverages, high initial counts of bacteria were obtained for the tested microorganisms, except *L. acidophilus*. In most of the samples, a statistically significant decrease in the viable population of the studied microorganisms was observed after 14 or 28 days of storage. However, this decrease did not go below 7 log10 CFU/mL in most of the tested samples for *Lactobacillus* sp., *S. thermophilus*, and *B. animalis* subsp. *lactis*. Tamang and Thapa [38] made the traditional Bhaati jaanr drink by fermenting an appropriately prepared rice beverage. After fermentation, the authors obtained viable counts of LAB (*Pediococcus pentosaceus* and *Loigolactobacillus bifermentans*, previously classified as *Lactobacillus bifermentans*) at a level of about 6 log CFU/g, and this value started to decrease from the seventh day of storage and reached approximately 5 log CFU/g on the 10th day. Other authors have also attempted to maintain viable counts of LAB during the storage period in fermented plant-based [39,40,41] and milk-based [42,43] beverages. Thus, it can be concluded that the survival of the initial number of bacteria involved in fermentation depends on the type of fermented product and the microorganisms used.

In general, the available literature lacks data on the changes in the population of PAB in fermented yogurt-type plant-based beverages. This may be due to the low use of these microorganisms in the fermentation of yogurt-type products. In the tested rice-based beverages, after fermentation, the viable population of *P. freudenreichii* subsp. *shermanii* in all samples was in the range of 4.80–5.56 log10 CFU/mL, and after 28 days of storage, the count decreased significantly in all samples to 3.40–4.60 log10 CFU/mL. Ranadheera et al. [44] fermented goat milk using various starter culture compositions, including novel putative probiotic *P. jensenii* 702. After fermentation, the authors determined the viable counts of *P. jensenii* 702 at 8.39 log10 CFU/mL, and this value did not decrease significantly during the 3-week period of storage under cooling. Zahed et al. [45] fermented skim milk and tested viable cells of microorganisms during 21 days of storage at 4 °C. They compared the fermentation process carried out using a combination of *P. freudenreichii* subsp. *shermanii* (PS4 starter culture) and LAB (*S. thermophilus* and *Lactobacillus delbrueckii* subsp. *bulgaricus*) and the process with LAB and inulin. After fermentation, the viable cell count of the starter cultures reached 9.18 log10 CFU/mL for the skim milk fermented with PS4 + LAB and 9.32 log10 CFU/mL for the milk fermented with PS4 + LAB + inulin. In both samples, the viable cell count of the starter cultures significantly reduced (to 5.98–6.33 log10 CFU/mL), but to a lesser extent for the samples fermented with added inulin. In the present study, it is shown that PAB can also be used in the fermentation of plant-based beverages, but to lower the pH of the product, they should be used in combination with LAB. Moreover, in the plant matrix, PAB reached a lower number than in the past research on the fermentation of dairy products. This may be due to the lower availability of simple carbohydrates in the plant matrix compared to the dairy matrix [26].

### 2.2. Physical Properties

**Figure 3:**
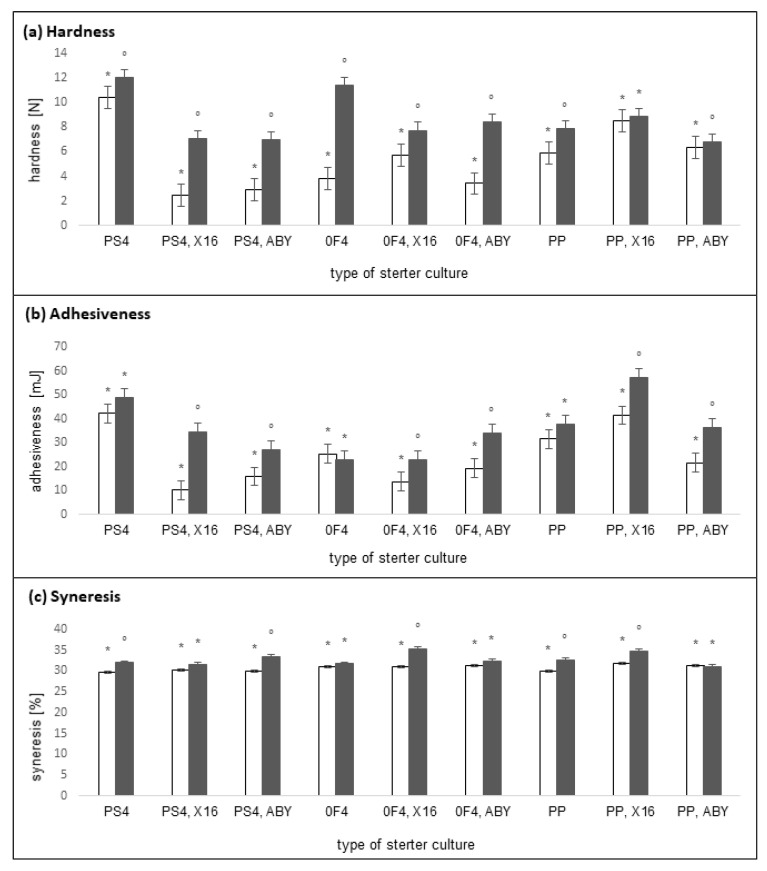
The changes in the physical properties (hardness, adhesive, syneresis) of rice-based beverages after 14 days of storage (white column), and after 28 days of storage (dark grey column) when inoculated with different starter cultures. Within each starter culture, the group means with a common symbol are not significantly different (*p* ≥ 0.05). Error bars represent standard error of the mean.

The changes during the 14- and 28-day storage period were assessed for 2 texture determinants (hardness and adhesiveness) and syneresis, and the obtained results are shown in Figure 3. The physical properties of yogurts are of great importance for their positive perception by consumers [46]. In the case of yogurt-type plant-based beverages, obtaining a texture similar to that of milk-based yogurt can be difficult due to the different compositions and structures of proteins [47]. Hardness is the peak force of the compression cycle, which is defined as the necessary force to achieve a required level of deformation. It is a critical texture property for yogurt-like products [48]. On the other hand, adhesiveness is the force required to detach a sample from the probe; it is necessary to separate the material that sticks to the teeth during eating [48]. Our analysis showed that the time of storage (*p* < 0.001) and type of starter cultures (*p* < 0.001) significantly influenced the hardness and adhesiveness of the tested beverages. However, there was no significant interaction effect (*p* = 0.204 for harness and *p* = 0.435 for adhesiveness). The hardness and adhesiveness of the tested samples after 14 days of storage were different depending on the starter culture used for fermentation. A significant increase in hardness was observed after 28 days of storage in all tested samples, except for the sample fermented with the PP–X16 (in which no significant changes were observed) and PP–ABY (in which a decrease in hardness was observed) combination of starter cultures. A significant increase in adhesiveness was also observed after 28 days of storage in all of the tested samples, except for the samples fermented with the use of starter cultures containing only *P. freudenreichii* subsp. *shermanii* (PS4, 0F4, PP), wherein no significant changes were observed. The fermentation carried out with each tested starter culture made it possible to obtain dense curd products. The available literature also lacks data on the effect of the time of refrigerated storage and the type of starter cultures on the hardness and adhesiveness of fermented yogurt-type plant-based beverages. These studies are performed mainly on milk-based yogurts and analyze the influence of LAB on the individual texture discriminants. Mani-López et al. [49] investigated the effect of selected probiotic LAB strains on the adhesiveness of yogurts or fermented milk. After 14 days of storage, the adhesiveness of the samples ranged from −0.002 to −0.112 N·s. After 28 days of storage, these values changed depending on the type of starter culture used. Increased adhesiveness was observed for samples fermented with starter cultures containing mixtures of *S. thermophilus* + *Lactobacillus bulgaricus* + *L. acidophilus*, *S. thermophilus* + *L. bulgaricus* + *Lactobacillus casei*, and *S. thermophilus* + *L. acidophilus*. Mousavi et al. [48] investigated the various texture determinants of yogurts fermented with *S. thermophilus* and *L. delbrueckii* subsp. *bulgaricus* without or with the addition of flaxseed. The hardness of the yogurt without the addition of flaxseed was 20.45 N and did not change during the 28-day storage period. The adhesiveness was at 31.26 N and decreased to about 18.5 N during storage. It is necessary to conduct analogous tests for the selected texture determinants in various types of yogurt-type plant-based beverages, including the influence of *Propionibacterium*.

Another important parameter for the physical quality of yogurt-type products is syneresis, which defines the release of serum from the gel matrix and is regarded as a technological defect in yogurt [50]. The syneresis test is often used to determine the degree of whey leakage from the yogurt. The degree of syneresis in fermented products affects their overall appearance and perception by consumers. The analysis showed that the time of storage (*p* < 0.001) and type of starter cultures (*p* < 0.001) significantly influenced the syneresis in the tested beverages and also had a significant interaction effect (*p* = 0.005). The syneresis value for all the samples remained high (>29%), as shown in Figure 3. Most samples exhibited an increase in the level of syneresis during the storage period, but not by more than 4.25%.

The study found that the level of syneresis ranged between 29% and 31% in all tested samples after 14 days of storage. After 28 days of storage, syneresis did not increase significantly. The available literature lacks data on the syneresis of fermented yogurt-type plant-based beverages and mostly focuses on the fermentation of yogurt with LAB. Mani-López et al. [49] investigated the effect of selected probiotic LAB strains on the syneresis of yogurts or fermented milk. They also tested the syneresis of two natural market yogurts, which was at 32.65% and 34.62%, respectively. All tested probiotic yogurts had values of syneresis >40%. Lower syneresis was observed during storage (by approximately 6–10%) for yogurts fermented with *L. delbrueckii* subsp. *bulgaricus*. After 14 days of storage, no significant differences were observed among the yogurts or fermented milk prepared with *S. thermophilus* + *L. delbrueckii* subsp. *bulgaricus* + *S. thermophilus* + *L. acidophilus* or with *S. thermophilus* + *L. reuteri*. Dan et al. [43] investigated the properties of fermented milk with two starter cultures: Lb-St-P8 (containing *Lactobacillus plantarum* P-8, *L. delbrueckii* subsp. *bulgaricus*, *S. thermophilus*) and Lb-St (containing *L. delbrueckii* subsp. *bulgaricus*, *S. thermophilus*). After 14 days of storage, the syneresis was at 33% for Lb-St-P8 fermented milk and 28% for Lb-St fermented milk. Considering the past literature results, the syneresis of the fermented rice-based beverages studied in the present work was at a favorable level, reaching values similar to or lower than that of milk-based yogurts.

Rice starch fermentation causes the disruption of starch crystallites and breakdown of starch granules. It also facilitates the swelling of starch molecules and enhances molecule interactions. This process affects the integrity of starch granules and the formation of rice gel matrixes [51] and results in the formation of a yogurt-type product with desirable textures and a relatively low degree of syneresis. However, the properties of rice starch are modified to a different extent during fermentation depending on the microorganisms used [51,52,53].

### 2.3. Carbohydrates Content

The changes in the content of the selected carbohydrates after 14 and 28 days of storage were examined in the rice-based beverages. Carbohydrates are used as substrates in the fermentation process with LAB. The analysis showed that the time of storage (*p* < 0.001) and type of starter cultures (*p* < 0.001) significantly influenced the content of fructose, glucose, maltose, sucrose, and raffinose in the tested beverages. They also had a significant interaction effect on the content of glucose, maltose, sucrose, and raffinose (*p* ≤ 0.001), but not on the fructose content (*p* = 0.198). The number of individual sugars in the tested samples varied depending on the starter culture used for fermentation. This indicates that different microbes make use of the rice carbohydrates to a different level.

**Figure 4:**
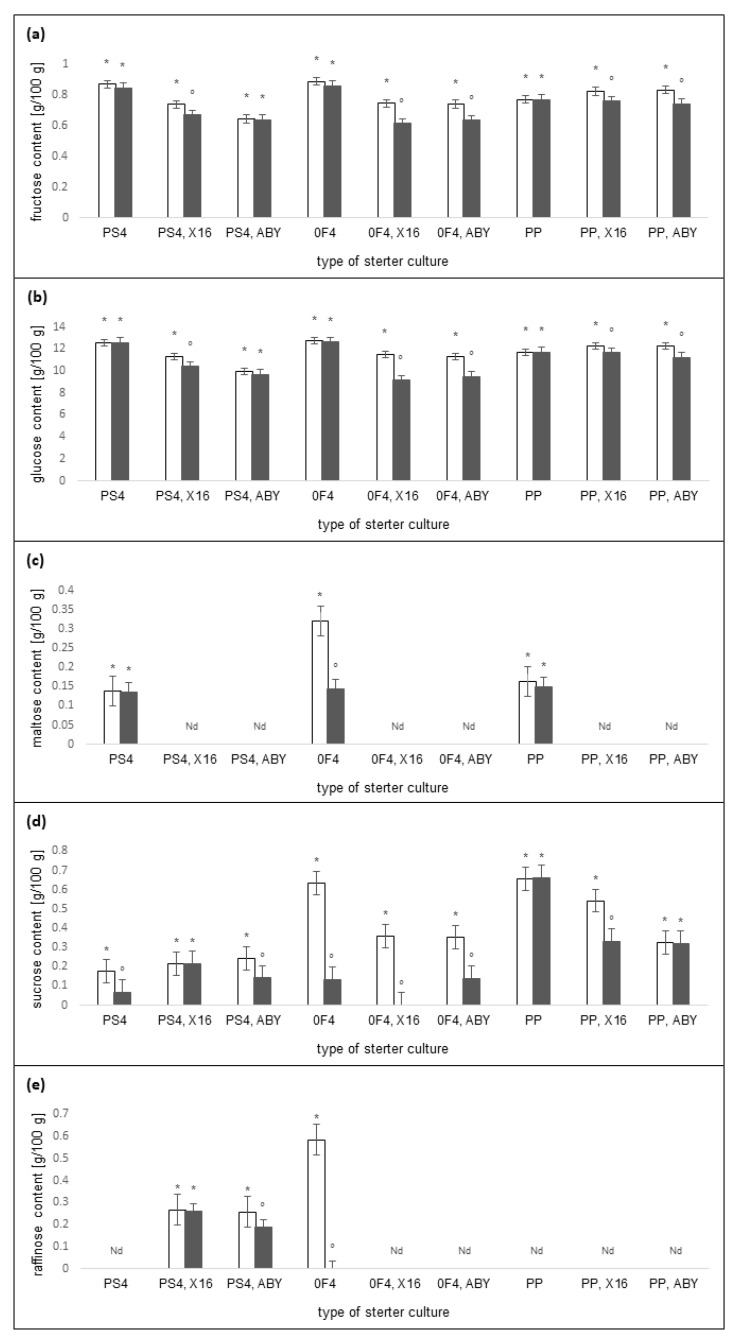
The changes in the (**a**) fructose, (**b**) glucose, (**c**) maltose, (**d**), sucrose, and (**e**) raffinose content in rice-based beverages after 14 days of storage (white column), and after 28 days of storage (dark grey column) when inoculated with different starter cultures. Within each starter culture, the group means with a common symbol are not significantly different (*p* ≥ 0.05). Error bars represent standard error of the mean. Nd—not detected.

During the fermentation of rice-based beverages, sugars are converted into metabolites, the nature of which depends on the type of microbes involved in the fermentation. LAB mainly use glucose as a carbon source during fermentation, while *Propionibacterium* mainly uses glucose, galactose, lactose, ribose, and fructose [54]. In all the samples after 14 days of storage, the highest glucose content ranged between 9923 and 12,733 g/100 g. Some samples showed a significant decrease in glucose content after 28 days of storage, but not below 9.155 g/100 g. The carbohydrate content of all the samples is presented in Figure 4.

In most of the samples tested, raffinose and maltose were not detected. These sugars may have been consumed by the microorganisms during fermentation. There is a lack of research on the individual carbohydrate content of fermented yogurt-type plant-based beverages. However, previous studies clearly show that during fermentation, the total carbohydrate content is significantly reduced [55,56]. Therefore, there is a need to study the changes in the content of individual carbohydrates in various types of plant-based beverages produced using LAB and PAB fermentation.

## 3. Materials and Methods

### 3.1. Preparation of Rice-Based Beverages

The rice-based beverages were prepared using rice flakes (Kupiec Sp. z o.o., Paprotnia, Poland) purchased from a local supermarket. According to the information on the packaging, the rice flakes had the following nutritional values (per 100 g of the product): energy value—351 kcal, fat—0.4 g (including saturated fatty acids—0.1 g), carbohydrates—77 g (including sugars—0.3 g), fiber—2.2 g, protein—8.8 g, and salt—0.02 g. The rice flakes were soaked in water in a 1:10 flakes-to-tap water ratio. The mixture was boiled for 5 min, as recommended by the manufacturer. After boiling, the mixture was weighed and supplemented with the evaporated water content. The mixture was blended and filtered through a sieve with a mesh size of 0.1 mm. The obtained beverage was supplemented with 10 g/L of glucose (Merck, Darmstadt, Germany). The beverages were poured into glass unit packages in portions weighing 140 g and were sterilized at 121 °C for 15 min.

### 3.2. Bacterial Strains

Five industrial freeze-dried starter cultures were used in the study. Two of them contained the typical microflora used in the production of fermented milk product, including the freeze-dried YC-X16 starter culture (Chr. Hansen, Hoersholm, Denmark) consisting of *S. thermophilus* and *L. delbrueckii* subsp. *bulgaricus*, and freeze-dried ABY-1 starter culture (Chr. Hansen, Hoersholm, Denmark) consisting of *S. thermophilus*, *L. delbrueckii* subsp. *bulgaricus*, *L. acidophilus* La-5, and *B. animalis* subsp. *lactis* Bb-12. The remaining three starter cultures contained only *P. freudenreichii* subsp. *shermanii*: freeze-dried *Propionibacterium* FD-DVS PS-4 FlavorControl™ (Chr. Hansen, Hoersholm, Denmark), freeze-dried *Propionibacterium* Propionici 000F0004 (Dalton Biotechnologie, Villa Raspa, Italy), and freeze-dried *Propionibacterium* PP (Biochem Srl., Montelibretti, Italy). The starter cultures were stored in a freezer at 18 °C until they were used in the fermentation.

### 3.3. Fermentation of Rice-Based Beverages

The inocula were prepared by dissolving the freeze-dried starter cultures in distilled water. The beverage samples were inoculated at 0.04% (m/m) and incubated at 45 °C for 6 h. After fermentation, the beverages were cooled at 6 °C and stored for 28 days for further analysis. The different combinations of starter cultures used in the fermentation and the sample codes are shown in Table 1.

### 3.4. Active Acidity and Microflora Analysis

The active acidity and microflora were analyzed before fermentation (immediately after inoculation), after fermentation, and after 14 and 28 days of refrigerated storage. The active acidity was determined by measuring the pH using a CPO-505 pH-meter (Elmetron, Zabrze, Poland). Measurements were made thrice for each sample.Microbiological analysis was performed using Petri dishes. De Man, Rogosa and Sharpe (MRS) agar medium (Merck, Darmstadt, Germany) was used to determine the viable population of *Lactobacillus* bacteria. The MRS-Clindamycin-Ciprofloxacin (MRS-CC) agar medium was prepared by modifying the MRS medium, which was sterilized, and then clindamycin (0.5 mL/L) and ciprofloxacin (5 mL/L) were added to inhibit the growth of LAB other than *L. acidophilus*. The MRS–CC agar medium was used to determine the viable population of *L. acidophilus* [57]. The M17 agar medium (BioMaxima, Lublin, Poland) was used to determine the viable population of *S. thermophilus*. The Bifidus Selective Medium (BSM) agar medium (Merck, Darmstadt, Germany) was used to determine the viable population of *B. animalis* subsp. *lactis*. The medium to determine the viable population of *P. freudenreichii* subsp. *shermanii* (hereinafter referred to as Propioni) contained yeast extract (10 g/L), Na_2_HPO_4_ × H_2_O (3 g/L), KH_2_PO_4_ (1 g/L) and sodium lactate (40 g/L) [58]. After dissolving the ingredients, the pH was adjusted to 6.97, the medium was poured into Schott bottles and 14 g/L of bacteriological agar (BTL, Warsaw, Poland) was added. All the media were prepared, sterilized (121 °C, 15 min), and poured into Petri dishes several days before inoculation. The Petri dishes with the prepared media were incubated at 37 °C for 2 days to allow them to dry and were then stored at 6 °C until analysis.

The drop plate method was used to determine the number of bacterial cells. A dilution series of the test samples was prepared, ranging from 10^−1^ to 10^−6^. The inoculation was introduced by placing 20 μL of each dilution (10^−6^ to 10^−2^) in the appropriately marked zones of the plates. The M17 agar plates were incubated at 37 °C for 168 h under aerobic conditions. The plates with MRS agar, MRS-CC agar, and BSM agar were incubated at 37 °C for 72 h and plates with Propioni agar for 168 h under anaerobic conditions. Anaerobic cultures were obtained in Anaerocult jars (Merck, Darmstadt, Germany) using Anaerocult™ A (Merck). After incubation, the number of grown colonies was counted and converted to CFU/mL. The result was expressed as a logarithm of the total cell count. The incubation was performed in three replications.

### 3.5. Texture Analysis

The texture analysis of the studied rice-based beverages included the determination of hardness and adhesiveness after 14 and 28 days of refrigerated storage. The tests were carried out using a Brookfield CT3 10 K texturometer (AMETEK Brookfield, MA, USA) with a TA4/1000 cylindrical probe (diameter: 38.1 mm, height: 20 mm). A pressure force of 0.04 N was applied during the experiment. The probe was moved at a speed of 2 mm/s toward the inside of the test sample and 4.5 mm/s in the opposite direction during withdrawal from the sample. The tests were carried out in three replications. The results were analyzed using the TexturePro CT V1.4 Build 17 software included with the measurement kit.

### 3.6. Syneresis Determination

The syneresis of the rice-based beverage samples was determined after 14 and 28 days of refrigerated storage. The analysis was performed using the MPW-350R centrifuge (MPW MED. INSTRUMENTS, Warsaw, Poland). The samples were mixed, weighed to 40 g, and centrifuged at 4 °C and 16,128× *g* for 30 min. After centrifugation, the supernatant was decanted and weighed. The value of syneresis, S (%), was calculated according to formula (1).

S = *A*/*B* × 100% (1)

where:

*A*—mass of the separated supernatant during centrifugation [g];
*B*—mass of rice-based beverage before centrifugation [g].

Measurements were made in three replications.

### 3.7. Carbohydrate Content Determination

The carbohydrate content of the rice-based beverages was determined after 14 and 28 days of refrigerated storage using high-performance liquid chromatography (HPLC). Initially, carbohydrates were extracted from the samples by mixing 8 g of the beverage and 32 g of methanol (Avantor Performance Materials Poland, Gliwice, Poland). The samples were mixed intensively, placed in an ultrasonic bath for 30 min at 30 °C, and then centrifuged in the MPW-350R centrifuge (MPW MED. INSTRUMENTS, Warsaw, Poland) at 4 °C and 16,000× *g* for 30 min. After centrifugation, the supernatant from the sediment was filtered through a syringe filter with a pore size of 0.45 µm (Merck, Darmstadt, Germany) and analyzed using HPLC. The analytes were separated using an HPLC kit equipped with DeltaChrom™ pumps, S 6020 Needle Injection Valve dosing loop (Sykam, Fürstenfeldbruck, Germany), DeltaChrom™ Temperature Control Unit column temperature controller (Sykam), and 05397-51 Cosmosil Sugar-D (4.6 ID × 250 mm, Cosmosil, Nacalai Tesque, Kyoto, Japan) column secured by Pre-column 05394-81 Cosmosil Guard Column Sugar-D (4.6 ID × 10 mm, Cosmosil). The analytes were detected using an S3580 RI (refractive index) detector (Sykam). Analyses were performed using isocratic elution. The mobile phase used was a mixture of acetonitrile (Avantor Performance Materials Poland, Gliwice, Poland) and deionized water in a ratio of 4:1. Sample solutions with a volume of 0.01 cm^3^ were dispensed into the system using a microsyringe. Each sample was analyzed for 30 min. Each sample was analyzed in triplicate. After the analysis, the peaks were identified by comparing the obtained retention times with that of the selected carbohydrate standards, including fructose, glucose, maltose, sucrose, and raffinose (Merck, Darmstadt, Germany). The content of selected carbohydrates in the beverage samples was calculated based on the area under the peak of the identified carbohydrate, considering the concentration degree of the initial sample.

### 3.8. Data Analysis

The study results were subjected to a two-way analysis of variance, using Statistica 13.1 (Dell Sp. z o.o., Warsaw, Poland). This helped determine the effect of the time and type of starter cultures used on the studied properties of rice-based beverages. The significance of the differences was analyzed using Tukey’s test at α = 0.05.

## 4. Conclusions

This study showed that the combination of lactic and propionic fermentation allows for the production of rice-based yogurt-type milk substitutes. The properties of the tested yogurts differed depending on the starter cultures used for fermentation. Fermentation using only *Propionibacterium* was insufficient to obtain a product with a similar level of acidity to that of milk-based yogurt. Rice-based yogurts were characterized by favorable physical properties, especially the syneresis degree, which was lower or close to that of milk-based yogurts. Propionic fermentation, which has so far been used mainly in the production of Swiss-type cheese, can also be used in the production of other dairy products. However, there is a need to expand the research on the properties of dairy products produced with the addition of PAB and to optimize their production technology.

## Figures and Tables

**Table 1:** Different combination of the addition of starter cultures used in the fermentation of rice-based beverages.

Starter Cultures	Codes
*Propionibacterium* FD-DVS PS-4 FlavorControlTM	PS4
*Propionibacterium* FD-DVS PS-4 FlavorControlTM + YC-X16	PS4, X16
*Propionibacterium* FD-DVS PS-4 FlavorControlTM + ABY-1	PS4, ABY
*Propionibacterium* Propionici 000F0004	0F4
*Propionibacterium* Propionici 000F0004 + YC-X16	0F4, X16
*Propionibacterium* Propionici 000F0004 + ABY-1	0F4, ABY
*Propionibacterium* PP	PP
*Propionibacterium* PP + YC-X16	PP, X16
*Propionibacterium* PP + ABY-1	PP, ABY

## Data Availability

Not applicable.

## Sample Availability

Samples of the compounds are not available from the authors.
